# Monitoring of the Sensitivity In Vivo of *Plasmodium falciparum* to Artemether-Lumefantrine in Mali

**DOI:** 10.3390/tropicalmed6010013

**Published:** 2021-01-24

**Authors:** Modibo Diarra, Drissa Coulibaly, Amadou Tapily, Boureima Guindo, Koualy Sanogo, Diakalia Koné, Youssouf Koné, Karim Koné, Aboudramane Bathily, Oumar Yattara, Mahamadou A. Thera, Alassane Dicko, Abdoulaye A. Djimdé, Issaka Sagara

**Affiliations:** 1 Malaria Research and Training Center, Department of Epidemiology of Parasitic Diseases, Faculty of Pharmacy, University of Sciences Techniques and Technology of Bamako, Bamako BP 1805, Mali; coulibalyd@icermali.org (D.C.); atapily@icermali.org (A.T.); bguindo@icermali.org (B.G.); koualy@icermali.org (K.S.); koneyoussouf155@gmail.com (Y.K.); konekeke86@gmail.com (K.K.); mthera@icermali.org (M.A.T.); adicko@icermali.org (A.D.); adjimde@icermali.org (A.A.D.); 2 National Malaria Control Program (PNLP-Mali), Bamako 223, Mali; dkone1311@yahoo.fr; 3 Populations Services International-Mali (PSI-Mali), Bamako E5397, Mali; abathily@psimali.org (A.B.); oyattara@psi.org (O.Y.)

**Keywords:** malaria, *Plasmodium falciparum*, artemether-lumefantrine, in vivo efficacy, Mali

## Abstract

In Mali, since 2007, artemether-lumefantrine has been the first choice against uncomplicated malaria. Despite its effectiveness, a rapid selection of markers of resistance to partner drugs has been documented. This work evaluated the treatment according to the World Health Organization’s standard 28-day treatment method. The primary endpoint was the clinical and parasitological response corrected by a polymerase chain reaction. It was more than 99.9 percent, the proportion of patients with anemia significantly decrease compared to baseline (*p* < 0.001), and no serious events were recorded. *Plasmodium falciparum* remains sensitive to artemether-lumefantrine in Mali.

## 1. Introduction

In the past two decades, we have witnessed a revolution in the diagnosis and treatment of malaria worldwide. In sub-Saharan Africa, classical antimalarial drugs have been gradually abandoned in favor of faster and more effective molecules, mainly because of the increasing resistance of parasites [1,2]. Indeed, artemisinin-based combination therapies (ACTs), recommended by the World Health Organization (WHO) since the beginning of the millennium [3], have been adopted in many African countries for the treatment of *Plasmodium falciparum* malaria, which has positively contributed to reducing the global burden of malaria [3].

In Mali, malaria remains a major health problem, with more than 2 million cases and more than 1050 deaths recorded in 2017, a case-fatality rate of 0.50‰ [4]. In addition to clinical cases and asymptomatic cases, malaria also contributes significantly to the occurrence of anemia, which has a particularly high prevalence in children under 5 years of age, with 82% and 51% in pregnant women according to the results of the Demographic and Health Survey of Mali (EDSM-V, 2012–2013). In the case of clinical malaria, the only way to avoid a fatal outcome remains rapid diagnosis and appropriate treatment. In this context, the case malaria management policy in Mali has opted for artemether-lumefantrine and artesunate-amodiaquine combinations as first lines drugs for the treatment of uncomplicated malaria [2] since the discontinuation of chloroquine therapy.

In order to promote evidence-based policy decisions, the WHO stressed the need to monitor regularly the efficacy of antimalarial drugs using standardized protocols [5].

In vivo efficacy of antimalarial as well as the evaluation of the prevalence of molecular markers associated with resistance of *Plasmodium falciparum* are essential to validate the treatment and guarantee a rapid response to the infection emergence of parasite resistance. In Mali, some studies showed a rapid selection of molecular markers of *P. falciparum* resistance to artemisinin partner drugs [2,6,7,8,9,10,11].

In Southeast Asia, reports have shown the emergence and potential spread of resistance to artemisinin [12,13,14], resulting in a significant reduction in the rate of parasite elimination in treated patients (half-life parasite clearance) with an artemisinin derivative alone or in combination with a partner molecule [15,16,17,18].

In an open and global world with the movement of populations, it seems appropriate to strengthen the monitoring of the effectiveness of ACTs. This study is part of the monitoring of the sensitivity of *Plasmodium falciparum* to artemether-lumefantrine in two sentinel sites of Mali’s National Malaria Control Program (NMCP) during two malaria transmission seasons 2017–2018.

## 2. Materials and Methods

Study sites and malaria in Mali: The study was conducted according to WHO standard in vivo efficacy protocol [19] in two NMCP sentinel sites, namely: (1) the Bougouni health district (Sikasso region); (2) Bandiagara Health District, (Mopti Region). In Mali, malaria transmission is seasonal. It spans from June to December, with a peak in October–November depending on the duration of the rain. The level of endemicity is also highly variable depending on the geographical area (Bougouni is in a Sudano-Guinean zone, and Bandiagara, a Sahelo-Sudanian zone. Both districts are intense and highly seasonal malaria transmission areas with longer transmission duration at Bougouni district).

The work took place in three community health centers in Bougouni district (Bougouni East, Bougouni West and Koumantou) and a district hospital in the Bandiagara district between July 2017 and December 2018.

Patients: The study population included patients aged six months or older with uncomplicated acute malaria confirmed by microscopy. Other inclusion criteria included body weight ≥5 kg, presence of fever (axillary ≥ 37.5 °C) or a history of fever in the previous 24 h, *Plasmodium falciparum* mono-infection with asexual blood density ≥1000/μL and <200,000/μL, and the absence of severe signs of complicated malaria as defined by WHO [20]. The main exclusion criteria included mixed malarial infections, hemoglobin level <5 g/dL, severe malnutrition, antimalarial treatment during the previous two weeks, ongoing prophylaxis in HIV-positive patients with cotrimoxazole or taking any other drug with antimalarial activity and any underlying serious illness. Patients meeting the inclusion criteria were enrolled if the parent/legal guardian signed a detailed written informed consent.

Treatment: Eligible patients were treated with Artemether Lumefantrine (each tablet containing 20 mg artemether and 120 mg lumefantrine). The study drug used was provided to the local health facilities by the Malian Ministry of Health. The dosage and method of administration of the drugs followed the manufacturer’s instructions and the Mali NMCP malaria treatment guideline. One dose at baseline (time 0), a second dose 8 h later, the third dose 24 h later and the last three doses 12 h apart (six doses in total) for 3 days. The number of tablets to be taken was determined according to body weight: one tablet for children from 5 to <15 kg, two tablets for children from 15 to <25 kg and three tablets for subjects of 25 to <35 kg and four tablets for subjects over 35 kg. Drug administrations were directly observed by the investigators. When vomiting occurred within 30 min of dosing, a new dose was re-administered. For febrile subjects (fever > 37.5 °C), paracetamol was used. In the event of signs of danger or severe malaria, the patient was hospitalized and given injectable artesunate, in accordance with the National Malaria Treatment Policy [3].

Follow-up: Follow-up visits took place on days 1, 2, 3, 7, 14, 21 and 28 after enrollment or at any time when the child was ill. Patients who withdrew their consent or participation were stopped prematurely for various reasons were followed through local guides until the end of the study. Adverse events that occurred were recorded, treated, and assessed by severity and drug-study relationship. Parasite clearance was monitored by microscopy. Microscopy slides were obtained and then stained with Giemsa later prior to each dose of AL and at each follow-up visit on days 2, 3, 7, 14, 21, and 28. The slides were examined by certified microscopists and were considered negative in the absence of parasites after examination of 200 fields in a thick smear of blood according to MRTC standard operating procedures. The parasite density was estimated by counting the number of asexual parasites in 200 white blood cells, assuming a standard count of 8000/μL. The determination of the species (and thus the confirmation of the mono-infection) was carried out on the basis of an evaluation of the thin films. As part of quality control measures, a second independent microscopist from the project team read 10% of all smears from all visits.

Dried blood spots (DBS) for Polymerase Chain Reaction (PCR) analysis were collected from each patient using 3MM Whatman™ filter papers at enrolment (day 0) and at 7, 14, 21 and 28 days, on the day of treatment failure or any other unplanned visits with suspected malaria. After drying, they were stored in plastic bags containing silica gel (desiccant) and were used to distinguish the recrudescence of a new infection using the procedures recommended by WHO [19]. For participants with recurrent parasitemia after day 7, paired dried blood spots (DBS) from day 0 and the day of parasite recurrence were genotyped for *Plasmodium* merozoite surface protein 2 genes (msp2) and microsatellite (CA1 and TA87) to discriminate reinfection from recrudescence as described previously [21,22,23]. Recrudescence was defined by at least one identical allele for each of the three markers in the pretreatment and posttreatment samples. New infections were diagnosed when all alleles of at least one of the markers differed between the two samples. Cases of new infections were excluded from the per-protocol analysis. A sequential approach was used to perform the PCR.

Study outcomes: The main measured outcome of the efficacy of AL was the adequate clinical and parasitological response (ACPR) at day 28 corrected by PCR. Then, early treatment failure (ETF), late clinical failure (LCF) and late parasitological failure (LPF) as defined by the WHO [19] were also determined. Secondary outcomes included uncorrected 28-day ACPR (crude efficacy), clearance of fever and gametocytes, tolerability, and changes in baseline hemoglobin at day 7 and day 28.

Data management and statistical analysis: The data were recorded using electronic case report forms with the open data kits software (ODK). All survey questionnaires were captured using a digital tablet and sent to a database hosted in a server at the Malaria Research and Training Center at the University of Science, Techniques and Technologies of Bamako (USTTB), Mali. Tablets were secured by an individual password and kept under seal outside working hours. Efficacy was calculated in the protocol-treated population, which includes all patients meeting the eligibility criteria of the protocol, having completed the three-day treatment of the study drug, having adhered to all study procedures until a possible failure or at the final evaluation of 28th day. Efficacy rates were calculated by dividing the number of patients with clinical and parasitological cure at day 28 by the total number of patients that could be assessed. In addition, statistical analyses (proportions, descriptive statistics or percentage comparison) were performed with the R version 3.4.3 software and the statistical significance level was set at 5%.

Sample size calculations: Previous studies in Mali found artemether-lumefantrine efficacy of at least 95% in the different research sites [24,25]. The sample size calculations were based on WHO methodologies [26] using a slightly more conservative expected efficiency estimate (95%) and a precision level of about 5%. To achieve this, and with a projected loss of follow-up rate of 20% by day 28, a minimum of 117 patients should be recruited, rounded to 120 subjects on each of the study sites.

Ethical considerations: The protocol was approved by the Ethics Committee of the Faculty of Medicine and Odonto-Stomatology and Pharmacy (FMOS-FAPH) of Mali before the start of all activities (Ref N° 2017/108/CE/FMPOS) and at the amendment for the extension of the study to 2018 (Ref N° 2018/108Bis/CE/FMOS). The trial was conducted in accordance with the guidelines of good clinical practice. Written informed consent was obtained from all participants. Informed assent was also obtained from children aged 12 to 17 years.

## 3. Results

### 3.1. Participant Flow

**Figure 1:**
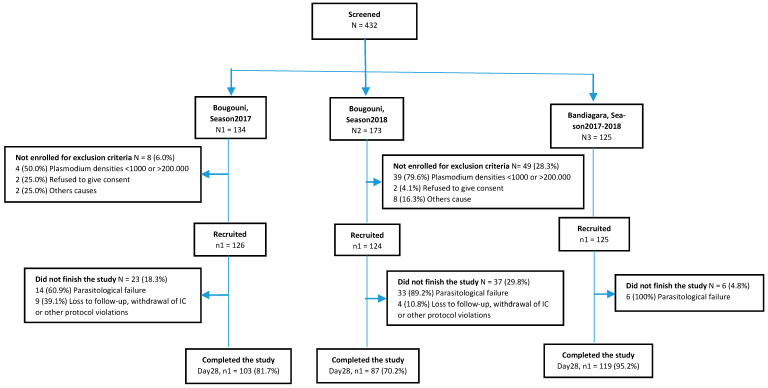
Study profile in both study sites during both seasons. * Parasitological failures are excluded as these were reinfections and not recrudescences.

Trial profile and baseline characteristics: A total of 432 febrile patients or patients with a history of fever within 24 h were screened between July 2017 and December 2018, of whom 375 (86.8%) were recruited and 330 (95.7%) completed the study, with or without recurrent parasitemia (Figure 1).

### 3.2. Baseline Data

The absence of *Plasmodium falciparum* mono-infection or the presence of danger signs such as vomiting or the notion of taking other antimalarials within two weeks or during follow-up and withdrawal of consent were the main causes of exclusion. Table 1 summarizes the basic characteristics of the two sites during the two seasons of the study.

### 3.3. Efficacy

**Figure 2:**
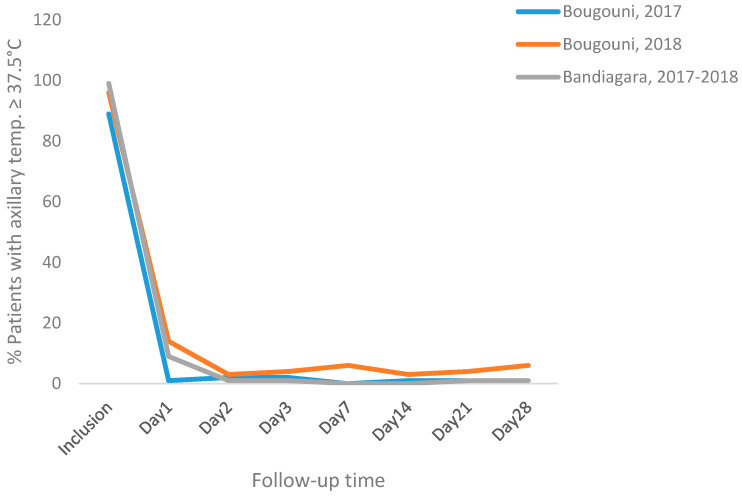
Proportion of patients with axillary temperature ≥37.5 °C in both sites during the 28-day follow-up.

During the 28-day follow-up during the two malaria transmission seasons 2017 and 2018, 13 patients (9 patients, 2017 season; 7.1% and 4 patients, 2018 season; 3.2%) did not successfully complete the study due to loss of follow-up, withdrawal of consent or other protocol violations such as the accidental or deliberate taking of antimalarial drugs, incoercible vomiting after inclusion or noncompliance with the visit schedule. Reported fever was the main recorded sign at the enrollment with 79.2% of the patients. This frequency decreased rapidly during the first 48 h of follow-up, to 2% or less throughout the rest of the follow-up (Figure 2).

The treatment results by locality according to season are summarized in Table 2. The cure rate not corrected by PCR on day 28 was 85.4% (309/362; 95% CI: 81.8–98.4). All 53 cases of recurrent parasitemia were found to be new infections according to PCR, resulting in a PCR-corrected cure rate of 100.0% (309/309) for the 28-day follow-up. Thus, the cure rate corrected by PCR on day 28 was identical at both study sites.

**Figure 3:**
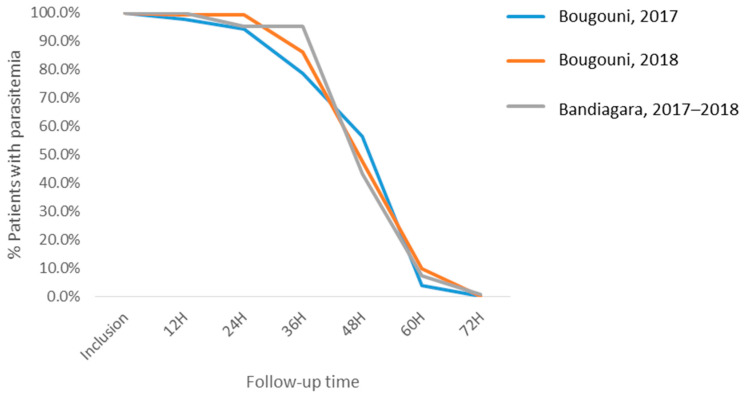
Proportion of patients with parasitemia in both sites during the first 72 h of follow-up.

For both locations, 50.9% of treated patients eliminated their parasitemia at the end of the first 48 h of follow-up, while 93.1% (22/439) did it at the end of the first 60 h of follow-up, and only 0.3% did not eliminate parasites at the end of the first 72 h of follow-up (Figure 3).

**Figure 4:**
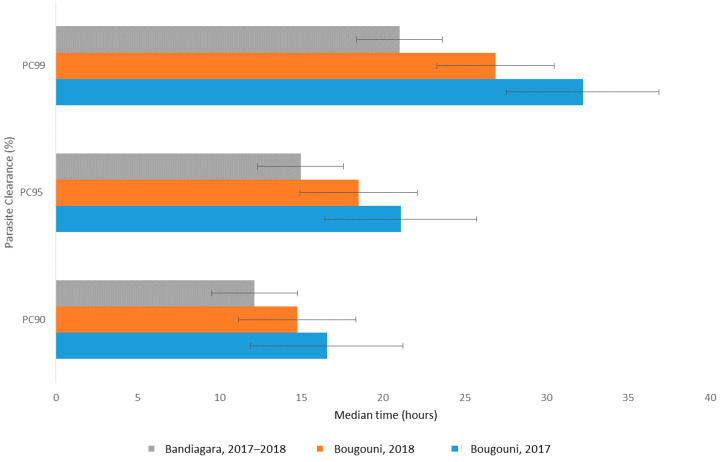
Median time required to eliminate 90%, 95% and 99% of parasitemia (hours) per site.

We also used a parasite clearance estimator from The Worldwide Antimalarial Resistance Network (WWARN) [27]. The estimated time in hours to reduce parasitemia by 99% of its initial value was 21.0 h for Bandiagara, 26.9 and 32.3 h for Bougouni in 2018 and 2017, respectively (Figure 4).

**Figure 5:**
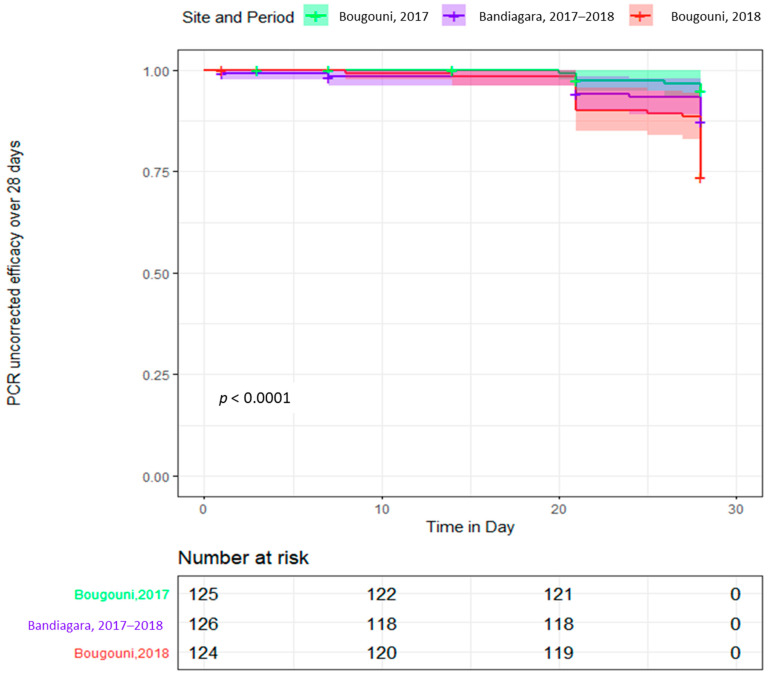
Kaplan–Meier survival curve showing artemether-lumefantrine PCR uncorrected efficacy over 28 days of follow-up period per study site and year.

The WHO indicated Kaplan–Meier survival analysis [26] assessing the uncorrected treatment efficacy showed a significant difference (*p* < 0.0001) between the study sites (Figure 5).

There was zero percent parasite recrudescence at all sites after molecular correction for reinfection.

The list of all the participants (n = 53) who had reinfection is provided in Appendix A.

### 3.4. Tolerability and Safety

Fourteen patients reported vomiting (14/375; 3.7%) during the three days of treatment in both locations (Table 3). Twenty-one episodes of abdominal pain (21/375; 5.6%), twelve episodes of headache (12/375; 3.2%) and eleven episodes of diarrhea (11/375; 2.9%) occurred in both locations; all of these events were transient and mild in nature (Table 3). These events could be related to malaria or the study drug.

The proportion of patients with hemoglobin below 11 g/dL at inclusion (94/375; 25.1%) was significantly improved on day 28 (60/330; 18.2%). This improvement was observed in all localities and in both seasons for the Bougouni site (Table 4). However, no improvement of hemoglobin was observed on day 7 of the follow-up compared to day 0. No serious adverse events (SEs) were documented in this study.

## 4. Discussion

This study describes the 28-day in vivo efficacy of AL under field conditions at two NMCP sentinel sites in Mali. These two sites, one Guinean Sudanese area, the other Sahelian area, provide a good geographical representation of the variability of malaria endemicity and transmission patterns in the country. The standard protocol for evaluating the in vivo efficacy of WHO ACTs was used at both sites [26], including molecular techniques for differentiation of new infections from recrudescences [19]. In this work, when recruiting patients, we have taken into account the realities on the ground to make it easier to achieve height by setting the inclusion parasitemia at 1000 trophozoite/µL of blood and extending recruitment to all patients who consulted the study centers. Despite these measures, the Bandiagara site took two transmission seasons to reach sample size. The other peculiarity of this work was the use of existing artemether-lumefantrine stocks in the health facilities where the study took place therefore the local storage conditions of the drugs that being used to treat malaria daily by the local health centers. Thus, artemether-lumefantrine provided by the local health facilities and used appeared to be efficacious, safe and well-tolerated as there were no serious adverse events, and the adverse events reported were minor and mostly unrelated to the study product. All episodes of vomiting occurred in patients with suspected vomiting after the first 30 min of treatment and therefore did not require dose re-administration except in two cases that were more likely to be drug rejections. No cases of repeated vomiting or changes in treatment have been reported. In addition, the occurrence of other adverse events during follow-up was rare. It also appeared that the proportion of patients with hemoglobin levels <11 g/dL was significantly reduced between enrollment and the end of the study at day 28. This trend was comparable to what other authors have described using artemether-lumefantrine [14]. On the other hand, seven days after the start of treatment, no effect was observed on the proportion of patients with hemoglobin ≥ 11 g/dL; rather, there was a significant trend in hemoglobin decrease.

From the point of view of efficacy, the cure rate corrected by PCR (>99.9%) remains high and adequate according to WHO recommendations. These results confirm those of previous studies in Mozambique and Myanmar, all of which have documented the efficacy and safety of the combination of artemether-lumefantrine [28,29].

The study was not designed to compare sites, so caution should be exercised in interpreting results such as late parasitological failure (LPF) levels, Es identified and the effect of treatment on hemoglobin recovery. Parasitic clearance was obtained at the sites within the first 72 h after treatment, apyrexia without analgesia was obtained 48 h after treatment as documented in 2016 in Mali [18], a reassuring sign regarding the issue of resistance raised by some authors [30,31].

Difficulties encountered were on the respect of the AL dose taking schedules, which often occurred late at night. In order to minimize the inconveniences linked to this situation, the investigators took the participants’ telephone number with their agreement and called them an hour or thirty minutes in advance or went directly to the participants’ homes with the help of the local guide if they encountered a problem in coming to the health center to take the doses. The same strategy was used for parasitological follow-up (every 12 h) until two consecutive negative thick blood smears were obtained. On the other hand, the 28-day follow-up time was also difficult because it was considered long and contributed to a relatively high number of people lost to follow-up.

The molecular genotyping with msp1, msp2 and The Glutamate-Rich Protein (GLURP) is commonly used to distinguish recrudescence from reinfection in the monitoring of antimalarial drug efficacy [32,33,34,35,36]. In this study, we have performed the genotyping on the 53 episodes of recurrent parasitemia to support their classification as new infections using msp2, PfCA1 and PfTA99 as usually performed here as described previously [21,22,23], which allows for the efficacy data comparison locally. The enrollment of participants with 1000 parasites/µL or above instead of 2000 parasites/µL or above may be a limitation of the study. However, we believe that this does not affect the validity of our results as malaria control strategies are rapidly changing the malaria epidemiology in malaria-endemic areas [3].

In addition, efforts are underway to perform the parasite molecular resistance of *Plasmodium falciparum* to antimalarial using the 53 episodes of recurrent parasitemia samples and the results will be published.

Continuous monitoring of the in vivo efficacy of artemether-lumefantrine across the country is necessary to detect early signs of decreased efficacy of artemether-lumefantrine.

## 5. Conclusions

This study showed high efficacy of AL against *Plasmodium falciparum* malaria after more than a decade of use of this drug and was safe and well-tolerated. The findings of this study advocate for the continuous use of AL as first-line therapy for uncomplicated malaria in Mali. However, a genotyping of the parasite molecular resistance to antimalarial is needed to support these findings, and the monitoring of AL efficacy should continue as recommended by WHO.

## Figures and Tables

**Table 1:** Basic characteristics of subjects enrolled according to the study season in both sites.

Parameters at Inclusion	Bougouni 2017 N = 126	Bougouni 2018 N = 124	Bandiagara 2017–2018 N = 125
Age (Year) Average (range)	10.6 (2–40)	11.0 (2–60)	11.5 (1–36)
Age groups, n (%)			
≤5 years	17 (15.6)	23 (18.5)	13 (10.4)
6–12 years	64 (58.7)	69 (55.6)	75 (60.0)
≥13 years	28 (25.7)	32 (25.8)	37 (29.6)
Sex, n (%)			
Male	39 (36.1)	57 (46.0)	35 (36.5)
Female	69 (63.9)	67 (54.0)	61 (63.5)
Average weight (kg) (range)	30.1 (6.0–96.3)	29.5 (8.6–83.1)	34.1 (10–90)
Average temperature (°C) (range)	37.6 (35.7–40)	38.2 (35.8–41.3)	38.2 (35.8–40.7)
Fever (≥37.5 °C), n (%)	89 (70.6)	96 (77.4)	99 (79.2)
Vomiting, n (%)	7 (5.6)	21 (16.9)	50 (40.0)
Parasite density geometric mean (range)	33.200 (1000–190.000)	50.426 (1040–199.320)	41.485 (1040–181.320)
Gametocytemia, n (%)	0	5 (4.0)	7 (5.6)
Average hemoglobin (g/dL), (range)	12.2 (9.1–16)	11.8 (8.1–17.7)	12.2 (8.0–16)
Anemia (Hb < 11 g/dL), n (%)	31 (24.6)	41 (33.1)	22 (17.6)

**Table 2:** Treatment outcomes on day 28, according to the study season in both sites in Mali.

Artemether-lumefantrine	Bougouni 2017	Bougouni 2018	Bandiagara 2017–2018	Total
Variable	N = 126	N = 124	N = 125	N = 375
ACPR ^a^ (uncorrected) n	103	87	119	309
ETF ^b^ n	0	0	0	0
LCF ^c^ n	0	0	0	0
LPF ^d^ n	14	33	6	53
New infections (with PCR) n	14	33	6	53
Recrudescences (with PCR) n	0	0	0	0
No treatment outcome (loss to follow-up or withdrawn) n	9	4	0	13
PP ^e^ day-28 efficacy (PCR-uncorrected) n/N (%, IC)	103/117 (88.0, 82.1–93.9)	87/120 (72.5, 64.5–80.5)	119/125 (95.0, 91.2–98.8)	309/362 (85.4, 81.8–98.4)
PP day-28 efficacy (PCR-corrected) n/N (%)	103/103 (100)	87/87 (100)	119/119 (100)	309/309 (100)

^a^ ACPR: adequate clinical and parasitological response; ^b^ ETF: early treatment failure; ^c^ LCF: late clinical failure; ^d^ LPF: late parasitological failure; ^e^ PP: per-protocol.

**Table 3:** Cumulative adverse events related to tolerability during the three days of treatment for both study sites in Mali.

Artemether-Lumefantrine	Study Sites			Total N = 375
	Bougouni, 2017 N = 126	Bougouni, 2018 N = 124	Bandiagara, 2017–2018 N = 125	
Vomiting post-dosing 1, 2 or 3 n (%)	1 (0.8)	4 (3.2)	9 (7.2)	14 (3.7)
Diarrhea n (%)	3 (2.4)	1 (0.8)	7 (5.6)	11 (2.9)
Abdominal pain n (%)	3 (2.4)	8 (6.5)	10 (8.0)	21 (5.6)
Headaches n (%)	0 (0)	8 (6.5)	4 (3.2)	12 (3.2)

**Table 4:** Proportion of patients with hemoglobin <11 g/dL between day 0 and day 28 in both sites.

Sites	Anemia		McNemar’s Test *p* Value
	Day 0	Day 28	
Bougouni, 2017 % (n/N)	24.6 (31/126)	16.0 (17/106)	<0.001
Bougouni, 2018 % (n/N)	33.1 (41/124)	28.8 (32/111)	<0.001
Bandiagara, 2017–2018 % (n/N)	17.6 (22/125)	9.7 (11/113)	<0.001
Total % (n/N)	25.1 (94/375)	18.2 (60/330)	<0.001

## Data Availability

Our study has one limitation in relation to the data availability policy. Indeed, this aspect was not taken into account during the process of obtaining informed consent from the study subjects nor during the process of obtaining ethics committee approval. In addition to this, the data on molecular markers of artemether-lumefantrine resistance will be specifically analyzed and the resulting results will be the subject of another manuscript.

## Appendix A

**Table A1:** Results of molecular analysis by study site and season.

ID Patient	Visit Days	Study Sites	Pfmsp2	PfCA1	PfTA99	Outcome
**Season 2017, Total: 14**						
BOS006	D0	Bougouni	A			1
BOS006	D21	Bougouni	B			
BOS009	D0	Bougouni	B			1
BOS009	D28	Bougouni	AC			
BOS017	D0	Bougouni	A			1
BOS017	D28	Bougouni	B			
BOS051	D0	Bougouni	A			1
BOS051	D28	Bougouni	BC			
BAD052	D0	Bandiagara	B			1
BAD052	UV	Bandiagara	A			
BAD049	D0	Bandiagara	A			1
BAD049	D21	Bandiagara	B			
BOS059	D0	Bougouni	A			1
BOS059	D28	Bougouni	B			
BOS075	D0	Bougouni	A			1
BOS075	D21	Bougouni	B			
BOS085	D0	Bougouni	A	C		1
BOS085	D28	Bougouni	A	AB		
BOS109	D0	Bougouni	A	A		1
BOS109	D28	Bougouni	A	B		
BOS114	D0	Bougouni	A			1
BOS114	D28	Bougouni	B			
BOS115	D0	Bougouni	AB			1
BOS115	D21	Bougouni	CD			
BOS129	D0	Bougouni	A			1
BOS129	D21	Bougouni	B			
BOS163	D0	Bougouni	AB	AB		1
BOS163	D21	Bougouni	A	C		
**Season 2018, Total: 39**						
BOK001	D0	Bougouni	A	B		1
BOK001	D28	Bougouni	9	A		
BOK002	D0	Bougouni	AC	A		1
BOK002	D21	Bougouni	AB	B		
BOK003	D0	Bougouni	A	A		1
BOK003	D21	Bougouni	BC	B		
BOK009	D0	Bougouni	AB	A		1
BOK009	D28	Bougouni	B	B		
BOK013	D0	Bougouni	AB	A		1
BOK013	D21	Bougouni	9	B		
BOK016	D0	Bougouni	A	A		1
BOK016	D28	Bougouni	9	B		
BOK019	D0	Bougouni	A			1
BOK019	D28	Bougouni	B			
BOK020	D0	Bougouni	AB	B		1
BOK020	D28	Bougouni	B	A		
BOK024	D0	Bougouni	A	A		1
BOK024	D28	Bougouni	9	B		
BOK025	D0	Bougouni	A	B		1
BOK025	D28	Bougouni	A	A		
BOK026	D0	Bougouni	A	B		1
BOK026	D28	Bougouni	B	A		
BOK028	D0	Bougouni	9	A		1
BOK028	D28	Bougouni	A	B		
BOK030	D0	Bougouni	B			1
BOK030	D21	Bougouni	A			
BOK031	D0	Bougouni	BD	AC		1
BOK031	D21	Bougouni	AC	B		
BOK038	D0	Bougouni	B	A	A	1
BOK038	D21	Bougouni	ABC	9	B	
BOK044	D0	Bougouni	B			1
BOK044	D28	Bougouni	A			
BOK048	D0	Bougouni	AB	9	A	1
BOK048	D28	Bougouni	A	A	B	
BOK050	D0	Bougouni	AB	A		1
BOK050	D21	Bougouni	C	B		
BOK053	D0	Bougouni	A	A	AC	1
BOK053	D14	Bougouni	9	A	B	
BOK058	D0	Bougouni	A			1
BOK058	D28	Bougouni	B			
BOK059	D0	Bougouni	A	A	A	1
BOK059	D28	Bougouni	A	B	B	
BOK060	D0	Bougouni	ACD	A		1
BOK060	D28	Bougouni	AB	B		
BOK062	D0	Bougouni	ABD			1
BOK062	D28	Bougouni	C			
BOO003	D0	Bougouni	AD	A		1
BOO003	D21	Bougouni	AB	B		
BOO029	D0	Bougouni	AC	B		1
BOO029	D21	Bougouni	B	A		
BOO018	D0	Bougouni	ABD	A		1
BOO018	D28	Bougouni	9	B		
BOO019	D0	Bougouni	C			1
BOO019	D28	Bougouni	AB			
BOO025	D0	Bougouni	AB	B		1
BOO025	D28	Bougouni	A	A		
BOO052	D0	Bougouni	AB	A	AC	1
BOO052	D28	Bougouni	9	A	B	
BOO045	D0	Bougouni	B			1
BOO045	D21	Bougouni	A			
BOO057	D0	Bougouni	A	B		1
BOO057	D28	Bougouni	AB	A		
BOO072	D0	Bougouni	AB	A	B	1
BOO072	D28	Bougouni	A	9	A	
BOO073	D0	Bougouni	AD			1
BOO073	D21	Bougouni	B			
BAD077	D0	Bandiagara	AB	A		1
BAD077	D21	Bandiagara	B	B		
BAD081	D0	Bandiagara	B	A	A	1
BAD081	D26	Bandiagara	AB	9	B	
BAD088	D0	Bandiagara	A	A		1
BAD088	D28	Bandiagara	AB	B		
BAD097	D0	Bandiagara	AB			1
BAD097	D21	Bandiagara	C			
BAD101	D0	Bandiagara	A			1
BAD101	D21	Bandiagara	CD			
BAD120	D0	Bandiagara	D			1
BAD120	D28	Bandiagara	A			

Table legend: letters A, B, C and D: band length. Number 9: no band.
